# Behavioral effects of SGK1 knockout in VTA and dopamine neurons

**DOI:** 10.1038/s41598-020-71681-9

**Published:** 2020-09-08

**Authors:** Marie A. Doyle, Ali R. Stark, Geza Fejes-Tóth, Aniko Náray-Fejes-Tóth, Michelle S. Mazei-Robison

**Affiliations:** 1 Neuroscience Program, Michigan State University, 766 Service Rd, ISTB 5017, East Lansing, MI 48824 USA; 2 Department of Molecular and Systems Biology, Dartmouth University, Hanover, USA; 3 Department of Physiology, Michigan State University, East Lansing, USA

**Keywords:** Neuroscience, Reward

## Abstract

Drugs of abuse cause significant neuroadaptations within the ventral tegmental area (VTA), with alterations in gene expression tied to changes in reward behavior. Serum- and glucocorticoid-inducible kinase 1 (SGK1) transcription, catalytic activity, and phosphorylation are upregulated in the VTA by chronic cocaine or morphine treatment, positioning SGK1 as a critical mediator of reward behavior. Using transgenic mouse models, we investigated the effect of SGK1 knockout in the VTA and in dopamine (DA) neurons to evaluate the necessity of protein expression for natural and drug reward behaviors. SGK1 knockdown in the VTA did not impact reward behaviors. Given VTA cellular heterogeneity, we also investigated a DA neuron-specific SGK1 knockout (KO). DA SGK1 KO significantly decreased body weight of adult mice as well as increased general locomotor activity; however, reward behaviors were similarly unaltered. Given that SGK1 mutants virally overexpressed in the VTA are capable of altering drug-associated behavior, our current results suggest that changes in SGK1 protein signaling may be distinct from expression. This work yields novel information on the impact of SGK1 deletion, critical for understanding the role of SGK1 signaling in the central nervous system and evaluating SGK1 as a potential therapeutic target for treatment of substance use disorders.

## Introduction

The abuse of illicit drugs is a substantial economic burden in the US^1^, yet treatments for addiction remain inadequate, in part due to our limited understanding of the drug-induced neuroadaptations underlying addiction. The mesocorticolimbic dopamine (DA) system plays a critical role in drug reward and addiction^2^. Chronic drug use induces neuroadaptations within the ventral tegmental area (VTA)^3^, with changes in gene expression and cell activity linked to addiction-like behaviors in mice^4,5^. Using RNA-sequencing, we previously identified serum- and glucocorticoid-inducible kinase 1 (SGK1) as one of the few genes transcriptionally upregulated in the VTA by both chronic psychostimulants (cocaine) and opiates (morphine)^6^, positioning SGK1 as a potential drug-induced mediator of reward behaviors.

Serum- and glucocorticoid-inducible kinase 1 (*sgk1*) is an immediate early gene originally discovered through its transcriptional regulation by serum and glucocorticoids^5^. This serine/threonine kinase is part of the AGC family of kinases, which also includes AKT and protein kinase C (PKC)^7^. Similar to these other AGC family members, maximal kinase activity is achieved through phosphorylation of the activation segment and hydrophobic motif: in SGK1, this occurs at Thr256 and Ser422, by 3-phosphoinositide-dependent kinase 1 (PDK1) and mammalian target of rapamycin complex 2 (mTORC2) respectively^8,9^. A unique phosphorylation site at Ser78 has also been identified and is regulated by extracellular signal-regulated kinase (ERK) signaling and epidermal growth factor (EGF) stimulation^10,11^. In the periphery, SGK1 has been well characterized for its ability to modulate the function of a broad range of ion channels and transcription factors^12^. Of note, whole body SGK1 knockout is not developmentally lethal, but mice show deficits in the regulation of salt excretion^13^ and impaired epithelial sodium channel processing^14^ when placed on a low salt diet. While SGK1 is widely expressed in the brain^6,15–21^, its protein-level regulation and functional role in the central nervous system remain poorly understood.

In the brain, *sgk1* gene expression is induced by virtually all classes of drugs of abuse in a variety of regions critical to drug reward behaviors^6,19,20,22,23^. However, much of this information was collected as part of large-scale screening studies and most did not include protein-level or behavioral assays. Therefore, the significance of gene regulation remains largely unexplored, though SGK1 is capable of modulating animal behavior. Specifically, in the prefrontal cortex (PFC) and hippocampus, viral-mediated overexpression of SGK1 mutants with reduced catalytic activity (T256A or S422A) or phospho-deficiency (S78A) were capable of impairing stress- and learning-induced behaviors^11,17,21,24–27^. Expanding on known drug-induced mRNA regulation, we previously identified increases in VTA SGK1 catalytic activity and phosphorylation at Ser78 following chronic, but not acute, cocaine or morphine administration^6^. Further, VTA overexpression of constitutively active SGK1 (S422D) altered locomotor sensitization to cocaine and morphine^6^, indicating a behaviorally relevant role for SGK1 signaling in drug-related behaviors.

Given the capacity for SGK1 catalytic activity and phosphorylation to regulate animal behavior as well as the induction of VTA SGK1 regulation by drugs of abuse, we sought to determine the effect of VTA SGK1 deletion on cocaine and morphine reward. Moreover, the VTA contains a diverse neuronal population composed of 60–65% DA, 30–35% gamma-aminobutyric acid (GABA), and 2–3% glutamate neurons^28–30^. Therefore, it is important to take neuronal cell type into account. Because VTA DA neuronal activity and output are critical modulators of drug reward^3^, we also investigated a DA neuron-specific SGK1 knockout. To investigate the effects of region- and cell type-specific SGK1 deletion on drug reward, novel VTA- and DA neuron-SGK1 knockout mouse models were utilized to assess altered cocaine reward via cocaine conditioned place preference (CPP) and morphine preference via morphine two-bottle choice task.

## Results

### Validation of VTA-specific SGK1 knockdown

**Figure 1:**
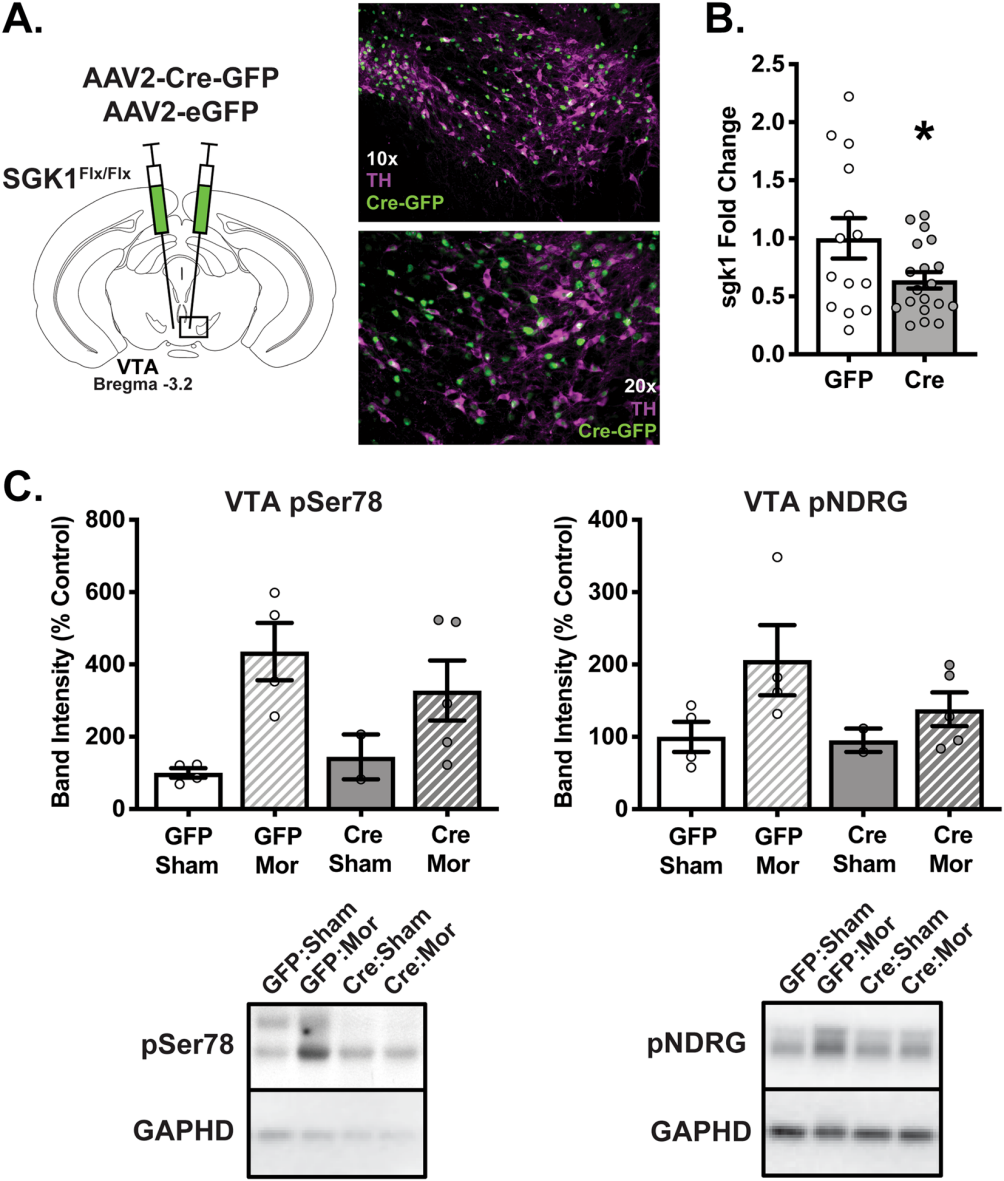
Establishment of VTA SGK1 knockdown mouse model. (**A**) Representative image of AAV2-Cre-GFP viral infection in VTA of FlxSGK1 mice at 10× and 20×. (**B**) VTA SGK1 knockdown (Cre) decreased VTA SGK1 mRNA expression compared to controls (GFP) (n = 14–19, unpaired t test, p = 0.0424). (**C**) Following morphine or sham treatment, there was a significant main effect of drug on VTA phospho-SGK1 (pSer78) and a trend on VTA catalytic activity (pNDRG) in GFP and Cre mice, though no main effect of virus was observed (pSer78 n = 2–5, two-way ANOVA followed by a Tukey post-hoc test, main effect of drug: p = 0.0067; pNDRG n = 2–5, two-way ANOVA, main effect of drug: p = 0.0565).

To create a conditional model of VTA-specific SGK1 deletion, male and female mice with homozygous floxed SGK1 gene (FlxSGK1) were bilaterally injected in VTA with AAV2-Cre-GFP to knockdown SGK1 expression within this specific brain region or with AAV2-eGFP as a vector control (Fig. 1A). Following surgery, VTA *sgk1* mRNA expression was significantly decreased in mice that received the Cre-recombinase (Cre) virus compared to GFP controls (Fig. 1B, all statistical analyses and results are detailed in Supplementary Table S1), confirming decreased gene expression. We next chronically treated a separate cohort of mice with subcutaneous morphine pellets in order to robustly induce SGK1 protein phosphorylation and activity as previously shown^6^. There was a main effect of drug on VTA phospho-SGK1 (pSer78) and a trend for a main effect of drug on VTA catalytic activity measured via phospho-N-myc downstream regulated gene (pNDRG, an exclusive target of SGK1^31^); however, there was not a significant effect of virus on VTA pSer78 and pNDRG (Fig. 1C). This lack of effect is likely due to the low number of mice, and additional cohorts are needed to confirm protein-level changes. Thus, intra-VTA AAV-Cre infusion resulted in knockdown of SGK1 gene expression, though an additional cohort is needed to validate protein-level reductions in VTA SGK1 catalytic activity and phosphorylation. Based on the significant decrease in SGK1 mRNA, we next sought to determine the behavioral consequences of reduced SGK1 expression within the VTA.

### VTA SGK1 knockdown does not alter natural or drug reward behavior

**Figure 2:**
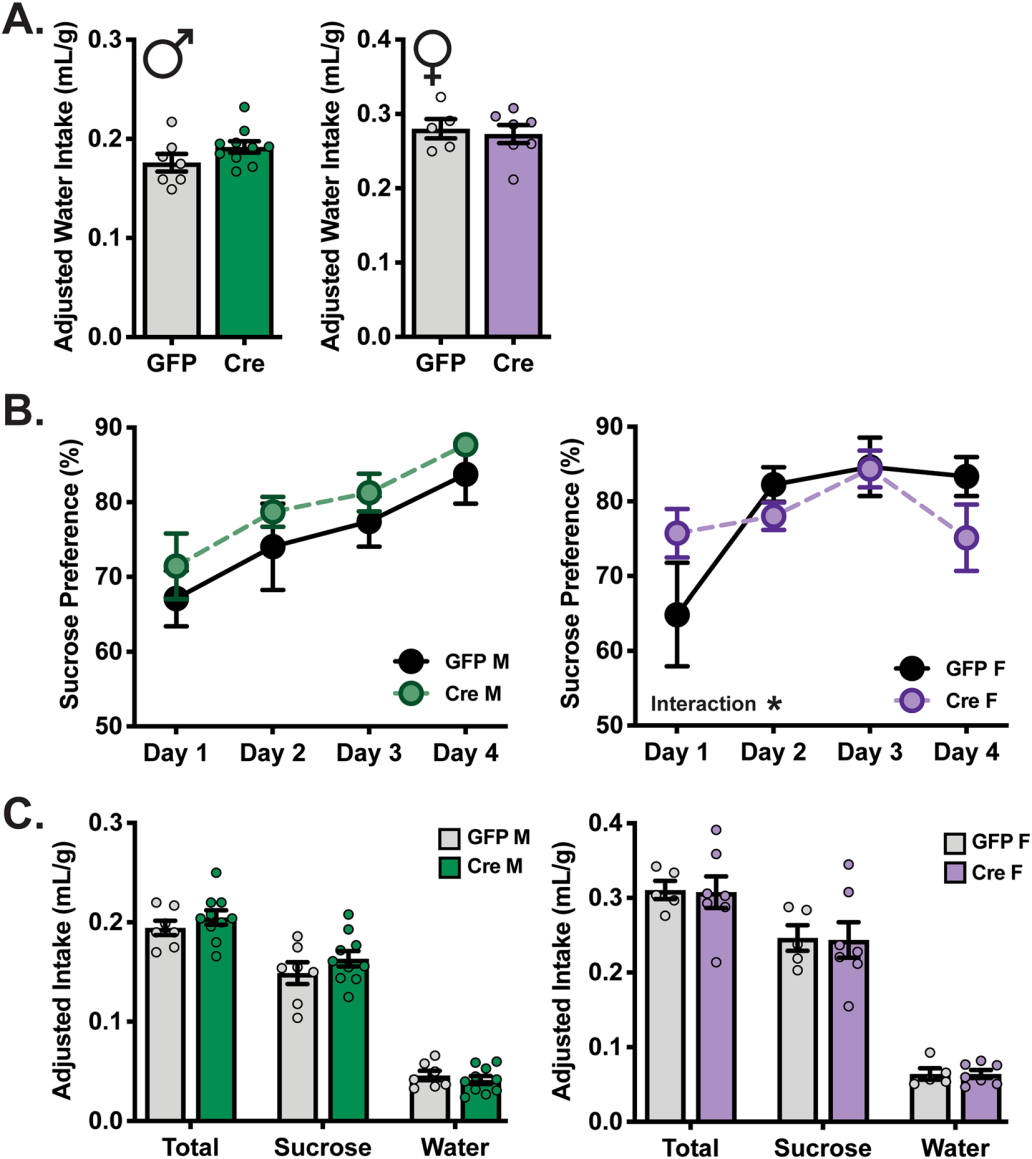
VTA SGK1 knockdown does not alter fluid intake and natural reward. (**A**) Water intake was not altered by VTA knockdown of SGK1 (male n = 7–10, unpaired t test; female n = 5–7, unpaired-test). (**B**) Natural reward measured via sucrose preference (1%) was not changed by VTA SGK1 expression (male n = 7–10, two-way ANOVA with repeated measures; female n = 5–7, two-way ANOVA with repeated measures, time x virus interaction p = 0.0126 but no significant effects in Sidak post-hoc test). (**C**) Intake of fluids is not altered by VTA SGK1 knockdown during sucrose preference test (male n = 7–10 unpaired t test; female n = 5–7, unpaired t test).

Because altered VTA gene expression is capable of disrupting natural reward^32,33^, we first sought to characterize potential changes in sucrose preference induced by VTA SGK1 deletion. Following viral surgery to knockdown VTA SGK1 expression, male and female mice were placed in a sucrose two-bottle choice (TBC) task to assess the impact of viral manipulation on sucrose preference. During acclimation to two water bottles, there was no significant difference between groups in total water intake, indicating that fluid intake was not altered by homozygous VTA SGK1 deletion (Fig. 2A). Natural reward was unchanged, as no difference was seen in preference for sucrose over the course of four days (average percent sucrose preference in males GFP:75.58 ± 3.33 Cre:79.79 ± 2.02 and females GFP:78.77 ± 3.06 Cre:78.31 ± 2.49) or average fluid intake during the task. There was a significant interaction of virus × time in female mice (Fig. 2B,C); however, a Sidak post-hoc test did not determine any significant differences between viral groups at any time-point. While interesting, this effect may have been driven by a low number of mice in the control group, and, therefore, an additional cohort of mice is needed to confirm this observation. These data were consistent with the fact that whole body SGK1 knockout mice do not display a phenotype until physiologically challenged (salt-restricted diet)^13,14^. Since acute drug treatment is not sufficient to induce significant changes in VTA SGK1 biochemistry^6^, natural rewards may not adequately engage SGK1 signaling pathways to drive deficits in reward behavior in our gene knockdown model.

**Figure 3:**
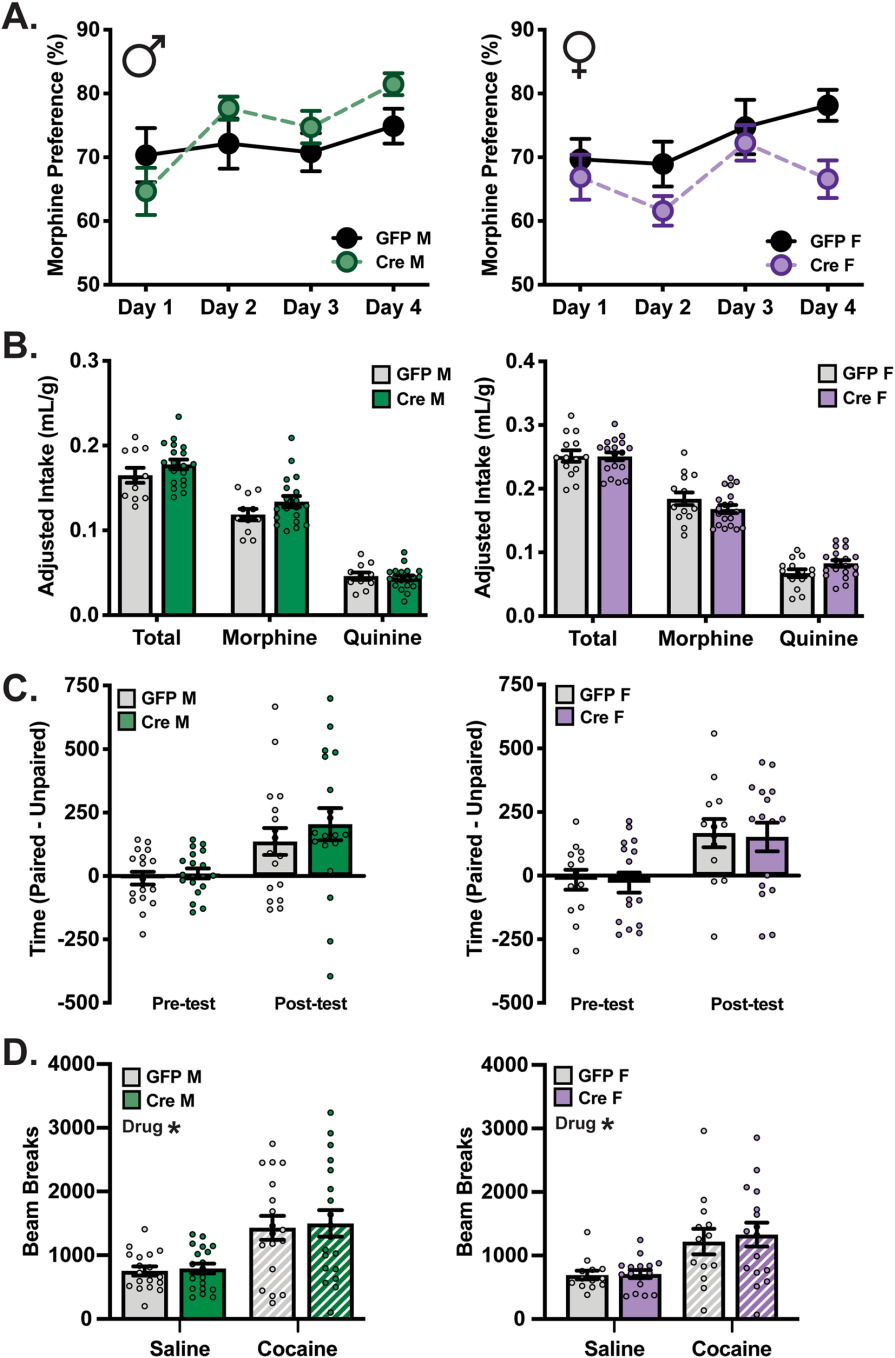
VTA SGK1 knockdown does not alter or morphine or cocaine reward behaviors. (**A**) Morphine preference was not significantly altered by VTA SGK1 knockdown (0.05 mg/mL morphine vs. 0.01 mg/mL quinine) (male n = 11–19, two-way ANOVA with repeated measures; female n = 14–19, two-way ANOVA with repeated measures). (**B**) Fluid intake between groups was not different during morphine TBC (male n = 11–19, unpaired t test; female n = 14–19, quinine: p = 0.0650). (**C**) Cocaine CPP in male (12.5 mg/kg) or female (10 mg/kg) mice was not altered by VTA SGK1 knockdown (male n = 18–19, unpaired t test; female n = 13–16, unpaired t test). (**D**) Two day average locomotor activity measured during saline and cocaine conditioning sessions was not different between VTA knockdown and control groups (male n = 18–19, two-way ANOVA followed by Tukey post-hoc, main effect of drug: p < 0.0001; female n = 13–16, two-way ANOVA followed by Tukey post-hoc, main effect of drug: p = 0.0002).

Given previously identified effects of chronic drug treatment on VTA SGK1 biochemistry and VTA constitutively active-SGK1 overexpression on drug reward behavior^6^, we next determined whether VTA SGK1 deletion caused a deficit in morphine preference in the TBC task and cocaine reward in conditioned place preference (CPP). Similar to sucrose reward, morphine preference was not significantly different in either male or female homozygous VTA SGK1 knockdown mice compared to GFP controls (Fig. 3A) (average percent morphine preference in males GFP:72.04 ± 2.10 Cre:74.64 ± 1.78 and females GFP:72.89 ± 2.50 Cre:66.83 ± 1.83; average morphine intake (mg/kg) in males GFP:5.93 ± 0.34 Cre:6.70 ± 0.33 and females GFP:9.22 ± 0.49 Cre:8.41 ± 0.32) nor in heterozygous knockdown males (Supplementary Fig. S1). Fluid intake during the task was not changed by VTA SGK1 knockdown in male or female mice (Fig. 3B).

SGK1 activity and phosphorylation were also increased by chronic cocaine treatment^6^, so we next examined the effects of altered VTA SGK1 expression on cocaine reward using CPP. Female mice were treated with a moderately lower dose of cocaine (10 mg/kg) compared to males (12.5 mg/kg) due to evidence for higher drug sensitivity for female rodents in CPP^34,35^. Following conditioning, neither male nor female homozygous VTA SGK1 knockdown mice showed a significant difference in time spent in the drug-paired chamber compared to controls, indicative of intact drug reward (Fig. 3C). Cocaine increased locomotor activity as expected, but SGK1 knockdown did not alter average total beam breaks between groups during either the saline or cocaine conditioning sessions (Fig. 3D). In all, VTA SGK1 knockdown did not appreciably alter reward behavior; however, while our viral expression was region-specific, it was not cell type-specific and knockdown in both VTA DA and GABA neurons could mask potential effects.

### DA neuron-specific SGK1 deletion alters body weight and locomotor activity

**Figure 4:**
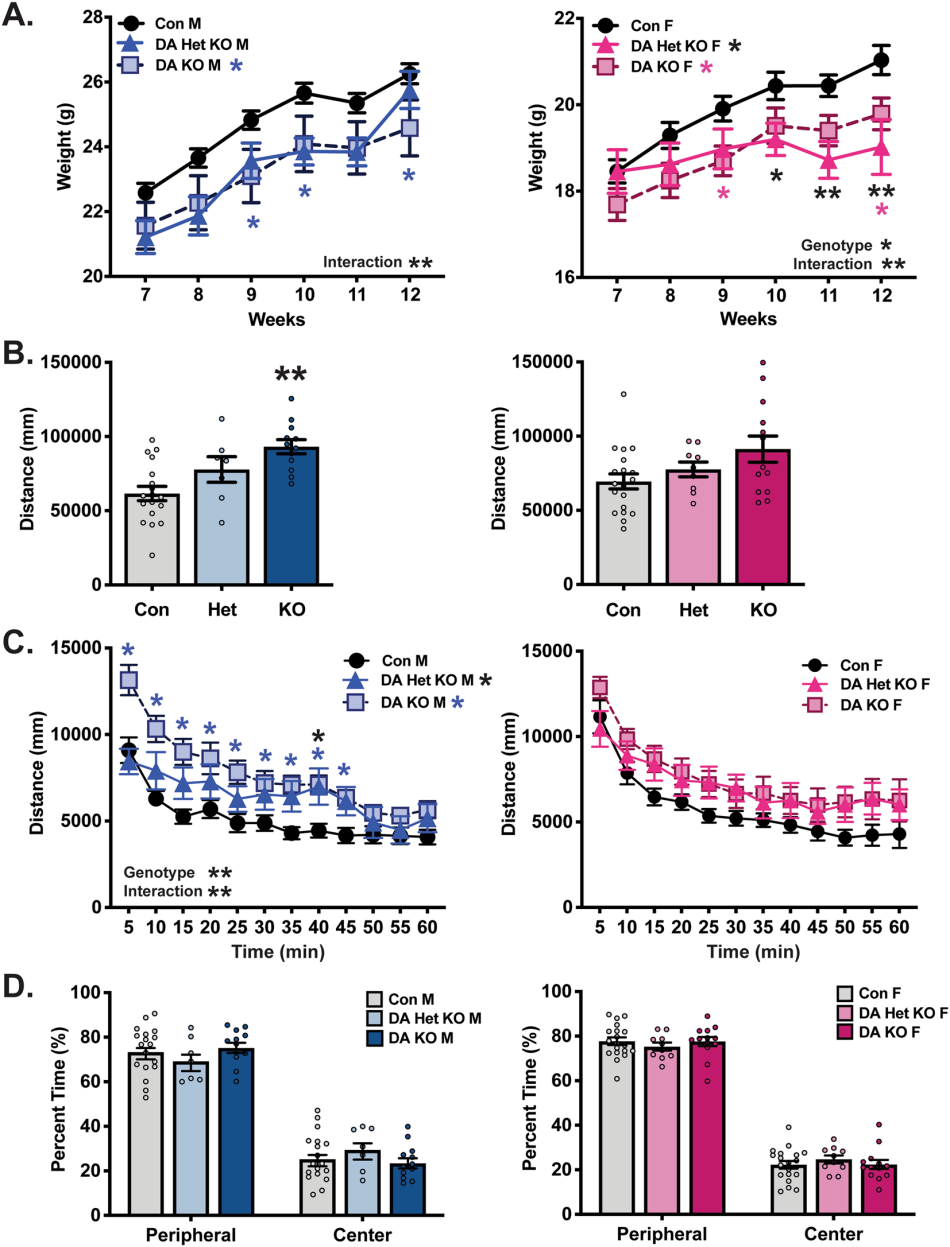
Body weight and locomotor activity are altered by DA SGK1 KO in both male and female mice. (**A**) There was a significant decrease in body weight of DA SGK1 KO mice compared to littermate controls over early adulthood in both males and females (7–12 weeks of age) (male con = 23, het = 7, KO = 12, two-way ANOVA with repeated measures followed by Sidak post-hoc, males time x genotype interaction: p = 0.0011 and main effect of genotype: p = 0.0566; female con = 19, het = 9, KO = 13, two-way ANOVA with repeated measures followed by Sidak post-hoc, time x genotype interaction: p < 0.0001 and main effect of genotype: p = 0.0328). (**B**) SGK1 knockout in DA neurons increased locomotor activity in an open field task in males (male con = 18, het = 7, KO = 12, one-way ANOVA followed by Dunnett post-hoc, main effect: p = 0.0006, Con vs KO: p = 0.0003; female con = 19, het = 9, KO = 13, one-way ANOVA, main effect: p = 0.0579). (**C**) Locomotor activity was similarly increased over time in a habituation curve for DA SGK1 KO males but not females (male con = 18, het = 7, KO = 12, two-way ANOVA with repeated measured followed by Dunnett post-hoc, main effect of genotype: p = 0.0006, time x genotype interaction: p < 0.0001; female con = 19, het = 9, KO = 13, two-way ANOVA with repeated measures). (**D**) During the open field task, there was no effect of DA KO on center time in male or female mice (male con = 18, het = 7, KO = 12, one-way ANOVA; female con = 19, het = 9, KO = 13, one-way ANOVA).

Given the neuronal heterogeneity in the VTA^28,29^ and the critical role for VTA DA activity and output in drug behavior^3^, we next evaluated the effects of SGK1 deletion in DA neurons. To exclusively delete expression from DA neurons, FlxSGK1 mice were crossed with dopamine transporter (DAT)-Cre mice to create a DA neuron-specific SGK1 deletion. Preliminary studies identified a significant interaction of morphine treatment and genotype on VTA phospho-SGK1 (pSer78), but additional cohorts are needed to further characterize protein-level changes in VTA SGK1 phosphorylation and catalytic activity (Supplementary Fig. S2). To determine if baseline physiology and behavior are altered by developmental DA SGK1 knockout (DA SGK1 KO), we first tracked body weight of heterozygous and homozygous DA SGK1 KO mice compared to littermates across early adulthood. Both sexes showed a significant time × genotype interaction, while females had an additional significant main effect of genotype (Fig. 4A). Overall, DA KO of SGK1 significantly decreased body weight of both male and female mice compared to their control littermates (Fig. 4A). Since homozygous DA SGK1 KO males showed a non-significant decrease in body length at 12 weeks of age (body length in centimeters Con:9.69 ± 0.11 KO:9.48 ± 0.07, n = 7–10), the effect of gene knockout on weight may be caused by a reduction in overall body size or a shift in metabolism, though additional cohorts would be required to confirm this observation. Locomotion is regulated in part by activity of DA neurons, so we next sought to determine if locomotor activity was altered in an open field task. Male mice had a significant main effect of genotype, where homozygous DA SGK1 KO mice displayed a robust increase in total distance traveled during this task (Fig. 4B, representative traces in Supplementary Fig. S3). In contrast, females did not display a significant increase in total locomotor activity (Fig. 4B). These effects were also seen in the habituation curve, where only male DA SGK1 KO locomotor activity was consistently higher than control littermates for the duration of the task (Fig. 4C). Neither sex showed a difference in center time during the open filed task, which may suggest that anxiety-like behaviors are unaltered by DA SGK1 KO (Fig. 4D). Overall, gene knockout of SGK1 in DA neurons caused a significant decrease in body weight in male and female mice and increased locomotor activity in males. We next sought to determine if reward-related behaviors were altered in this cell type-specific model.

### Reward behaviors, both natural and drug, are not changed in DA SGK1 KO mice

**Figure 5:**
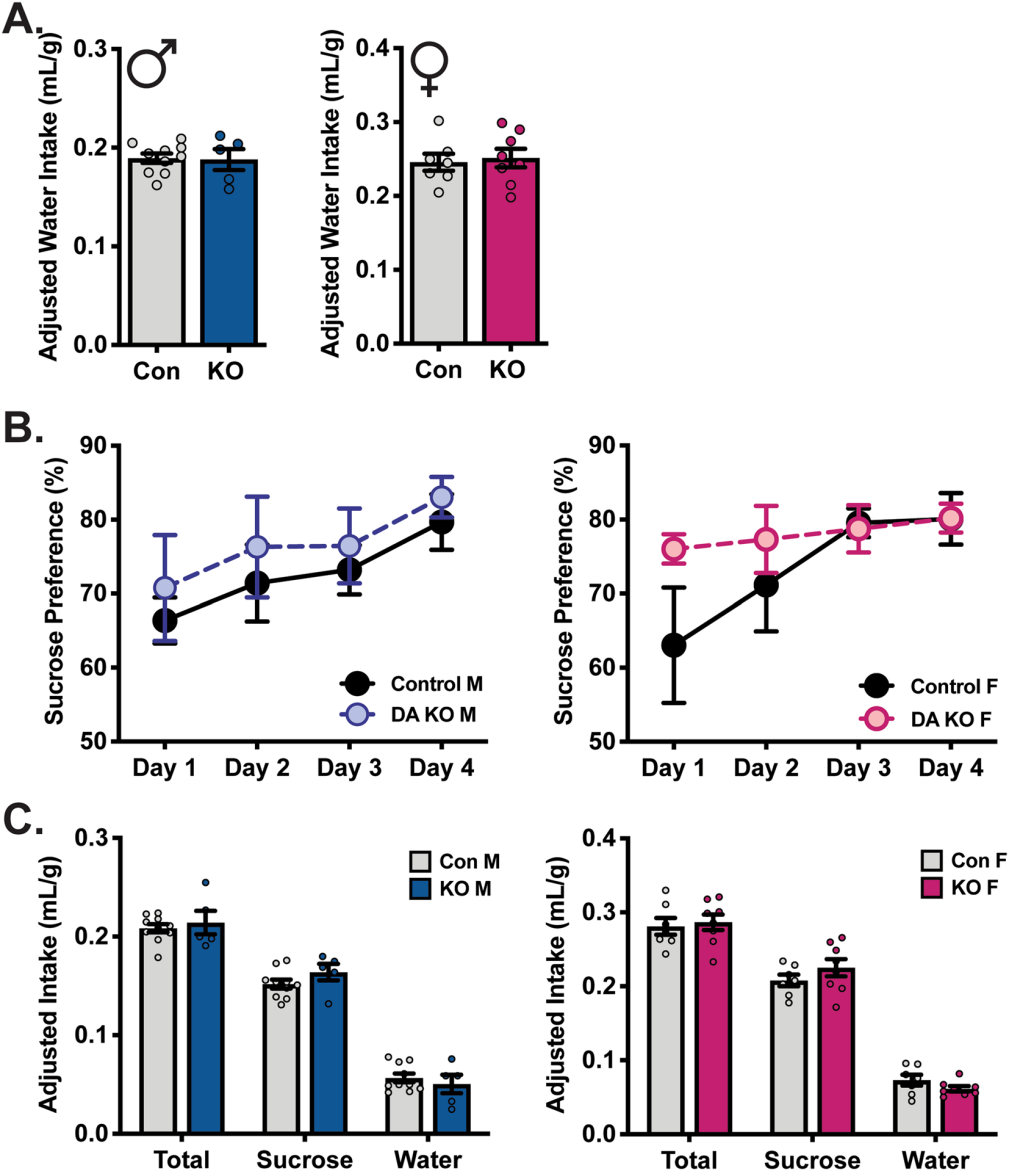
Fluid intake and preference for natural reward is not altered by DA SGK1 KO. (**A**) Water intake was not altered by DA SGK1 KO (males n = 9–13, unpaired t test; females n = 9–12, unpaired t test). (**B**) No differences in sucrose preference (1%) were seen in DA KO mice compared to littermate controls (male n = 5–10, two-way ANOVA with repeated measures; female n = 7–8, two-way ANOVA with repeated measures). (**C**) Average intake volumes were not altered during sucrose TBC task across genotype or sex (male n = 5–10, unpaired t test; female n = 7–8, unpaired t test).

As DA is a critical mediator of reward behavior^3^, we first examined altered natural reward in homozygous DA SGK1 KO mice. To test this behavior, we again used the sucrose TBC task and observed no difference in fluid intake during water bottle acclimation (Fig. 5A). Parallel to the VTA-specific SGK1 knockdown model, DA SGK1 KO mice showed no difference in sucrose preference over four days (average percent sucrose preference in males Con:72.67 ± 1.88 KO:76.64 ± 3.70 and females Con:73.46 ± 2.10 KO:78.07 ± 1.59) or average fluid intake during the task (Fig. 5B,C).

**Figure 6:**
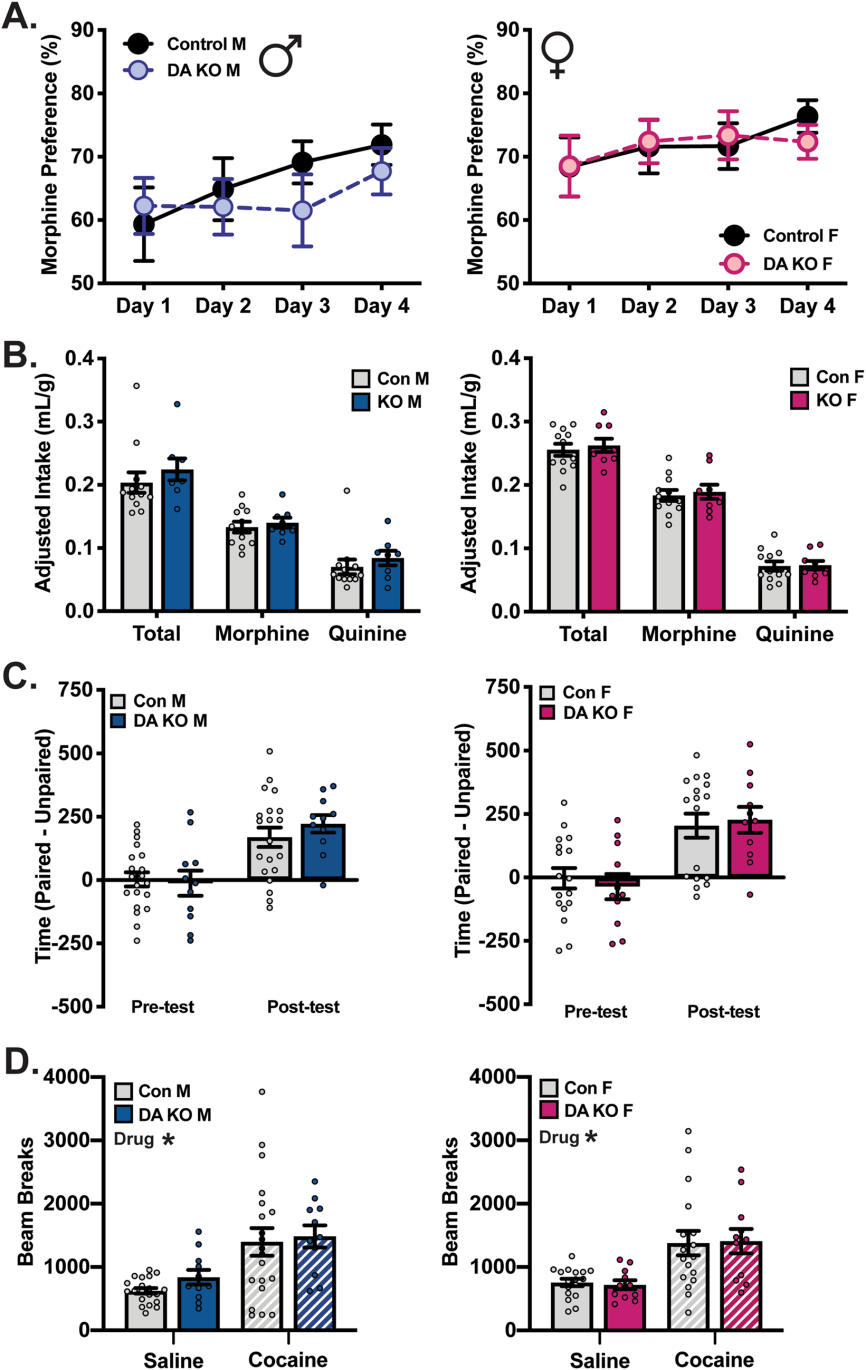
DA SGK1 KO does not alter morphine or cocaine reward. (**A**) Morphine preference (0.05 mg/mL morphine vs. 0.01 mg/mL quinine) was not altered by DA SGK1 KO (male n = 8–12, two-way ANOVA with repeated measure; female n = 9–12, two-way ANOVA with repeated measures). (**B**) Intake of fluids was not changed by genotype during morphine TBC task (male n = 8–12, unpaired t test; female n = 9–12, unpaired t test). (**C**) Cocaine reward measured by conditioned place preference was not altered in male (12.5 mg/kg) or female (10 mg/kg) mice (males n = 11–20, unpaired t test; females n = 11–17, unpaired t test). (**D**) There was not a main effect of genotype on locomotor activity during CPP conditioning sessions (males n = 11–20, two-way ANOVA followed by Tukey post-hoc, males main effect of drug: p = 0.0001; females n = 11–17, two-way ANOVA followed by Tukey post-hoc, main effect of drug: p < 0.0001).

To observe morphine and cocaine reward behaviors, we utilized morphine TBC and cocaine CPP, as in previous experiments. First, in the morphine TBC task, there was no effect of homozygous DA SGK1 KO on morphine preference (average percent morphine preference in males Con:66.31 ± 2.68 KO:63.63 ± 2.97 and females Con:72.01 ± 2.44 KO:71.68 ± 2.57; average morphine intake (mg/kg) in males Con:6.66 ± 0.42 KO:7.00 ± 0.40 and females Con:9.17 ± 0.43 KO:9.46 ± 0.56) or average fluid intake (Fig. 6A,B), consistent with VTA-specific SGK1 knockdown (Fig. 3A,B). In the CPP task, neither male nor female homozygous DA SGK1 KO mice differed from littermate controls in time spent in the cocaine-paired chamber following conditioning, indicative of intact drug (Fig. 6C). Locomotor activity during the conditioning sessions showed an overall main effect of drug treatment for both male and female mice, but no significant effects were observed between genotypes (Fig. 6D). These data indicate that while DA SGK1 KO modestly altered baseline measurements of body weight and locomotor activity in an open field assay, behaviors related to natural and drug reward, as assessed by TBC and CPP, were not impaired by SGK1 knockout.

## Discussion

The main objective of these studies was to characterize the effect of VTA- and DA neuron-specific SGK1 gene deletion on drug-related behaviors. Since VTA SGK1 mRNA, catalytic activity, and phosphorylation are upregulated by chronic cocaine and morphine treatment^6^, we predicted that drug reward-related behaviors would be particularly vulnerable to the effects of SGK1 deletion; however, neither VTA knockdown nor DA neuron-specific deletion was sufficient to disrupt morphine preference or cocaine reward. These data may suggest that SGK1 knockout does not directly affect drug reward behaviors. Other studies have identified stimuli-induced changes in VTA gene regulation or function without subsequent effects on behavior. For example, decreased VTA dopamine receptor 2 (D_2_R) expression and sensitivity has been observed following psychostimulant self-administration^36,37^; however, VTA knockdown of D_2_R did not alter the number of sucrose or cocaine rewards earned during operant self-administration, though D_2_R knockdown did increase motivation to earn rewards when rats were tested under a progressive ratio^38^. Similarly, chronic social defeat stress has been shown to decrease AKT phosphorylation in the VTA and overexpression of AKT mutants alters stress susceptibility^39^, however genetic knockdown of the kinase responsible for AKT phosphorylation did not increase susceptibility to stress^40^. Together these studies demonstrate the potential for disconnect between stimuli-induced gene and protein regulation in the VTA and effects of experimenter manipulation.

However, as SGK1 shares overlapping substrates with other AGC family kinases, opportunistic compensation may also explain a lack of observable effect in our models^41–45^. Of note, opportunistic compensation of AKT and SGK1 has been characterized in breast cancer cells^46^. It is possible that a similar method of compensation occurred in our novel neuronal models, masking potential effects. Alternatively, compensation may have occurred at a circuit level. SGK1 is capable of regulating a variety of ion channels and transporters in the periphery^12^, but the role of SGK1 in the brain is poorly understood. The electrophysiological effects of SGK1 KO are unknown in the VTA or in DA neurons and remain an area of interest. Further research is needed to determine if molecular or circuit level compensation is protective of SGK1 KO effects.

While SGK1 expression itself was not required for these drug-reward behaviors, recent work has highlighted the functional requirement for SGK1 catalytic activity and phosphorylation, where experimental manipulation of SGK1 catalytic activity and phosphorylation in the PFC^17,27^ and hippocampus^21,24–26,47^ have been linked to altered stress- and learning-induced behavior. Specifically, in the PFC, viral-mediated overexpression of a catalytically inactive SGK1 mutant (S422A) increased escape failures during a learned helplessness task^17^. Similarly, SGK1 protein interference in the prefrontal cortex via infusion of TAT-SGK1 peptides prevented stress-enhanced performance in a T-maze^27^. Both cases illustrate SGK1’s ability to orchestrate behavior in rodent models of stress. In the hippocampus, catalytically inactive SGK1 (S422A) overexpression impaired escape latency in the Morris water maze^21,26^ while phospho-deficient (S78A) overexpression decreased retention of contextual fear conditioning^24^, suggesting that hippocampal SGK1 is functionally required for these learning tasks. These viral-mediated overexpression and protein interference methods for assessing the role of SGK1 protein function may be fundamentally different from KO models. The presence of SGK1 protein in these models, both endogenous and overexpressed, may not have engaged opportunistic compensation pathways, such that deficiencies in SGK1 function related to catalytic activity and phosphorylation were capable of impacting behavior where our gene knockouts were not.

Despite a lack of drug reward effects, developmental DA SGK1 KO caused a significant decrease in body weight in male and female mice and increased open field locomotor activity in males. While changes in locomotor activity were not seen with our conditional VTA-specific knockdown, there were key differences between the two models, namely timing and region-specificity of knockdown. First, our models differed in adult vs. developmental SGK1 gene deletion. The decreased body weight and increased activity found in the male DA SGK1 KO mice is consistent with literature demonstrating that developmental modification of the DA system causes changes in weight and locomotor activity. Homozygous dopamine transporter (DAT) KO mice showed decreased body weight and robustly increased locomotor activity in an open field assay^48^. Additionally, this developmental model displayed significant adaptations in DA homeostasis as a result of gene deletion^48,49^. Altered homeostasis is a caveat of developmental models that is important to keep in mind for the interpretation of these data.

Secondly, though the VTA represents a well characterized region of DA neurons in the brain, DA neurons are expressed in other regions, including the substantia nigra, hypothalamus, periaqueductal gray, and olfactory bulbs^50,51^. The DA SGK1 KO mice used in these studies did not have gene deletion confined to the VTA, so SGK1 KO in another dopaminergic region may have contributed to the observed effects on locomotor behavior and weight. For example, DA signaling from the substantia nigra has been characterized as a critical mediator of locomotor activity^52,53^. Thus, it is possible that SGK1 KO in this dopaminergic population contributed to the observed increase in open field locomotor activity in our model. Moreover, stimulation of TH-positive (DA-producing) neurons in the arcuate nucleus has been shown to increase food intake while silencing these neurons reduces body weight, suggesting that SGK1 deletion in hypothalamic DA neurons could contribute to effects on metabolism^54^.

Further, the modest effects found in both our models were consistent with the phenotype of whole body SGK1 KO mice, which showed only mild physiological effects at baseline^13,14^. Instead, physiological challenges are required to show deficits, examples of which have been best characterized in the renal system. Whole body SGK1 KO mice were viable and largely comparable to WT mice at baseline; however, when mice were placed on a salt-restricted diet, KO mice showed impaired salt retention in response to the change in diet^13^, which was linked to disrupted epithelial sodium channel (ENaC) processing^14^. Given mild KO effects compounded with the fact that acute cocaine or morphine treatment does not induce changes in VTA SGK1 catalytic activity or phosphorylation ^6^, more chronic paradigms may be needed to assess reward behavior following long-term drug administration such as locomotor sensitization or self-administration. It is also possible that higher drug concentrations could expose effects of genetic SGK1 deletion on CPP or TBC. We chose the doses used in this study based on their ability to produce stable, but not maximal, cocaine CPP and morphine preference. While we predicted that genetic SGK1 deletion would decrease CPP and TBC, we did not want to discount the possibility that these measures may be increased by SGK1 manipulation, thus we chose not to use drug doses that might lead to a ceiling-effect. Though our data do not support effects of SGK1 genetic deletion on CPP and TBC, these could be limited by the experimental design. For example, performing additional cocaine conditioning sessions can increase CPP expression^55,56^; it is possible that SGK1 expression may be necessary to produce maximal CPP expression without impairing the ability to form a CPP. Similarly, there are limitations to the TBC task used to assess morphine preference. One caveat is the use of quinine, which is itself, aversive. It is possible that SGK1 KD/KO could produce opposite effects on morphine reward and quinine aversion, thereby obscuring an overall effect in the morphine TBC assay. These experimental considerations, as well as the possibility for opportunistic compensation of kinase activity, leave open the possibility that VTA or DA SGK1 protein activity could affect aspects of drug behavior under different conditions, but our current findings that SGK1 expression in VTA or DA neurons is not required to establish a CPP or preference in TBC remain.

In summary, our data suggest that SGK1 expression in the VTA or in DA neurons is not required for early stages of drug reward or intake. Strategies that utilize mutant-SGK1 overexpression or interfere with the function of endogenous protein expression may be necessary to uncover the role of SGK1 in the context of drug-related behaviors. Altogether, this work provides novel information about SGK1 expression and builds on a limited knowledgebase for the function of SGK1 in the central nervous system, both necessary to understanding the impact of SGK1 regulation on drug-reward behaviors.

## Methods

### Mice

Initial floxed SGK1 (FlxSGK1) breeder mice^14^ were obtained from A. Náray-Fejes-Tóth (Dartmouth University) and bred homozygously (SGK1^Flx/Flx^) for VTA SGK1 knockout experiments. To generate dopamine SGK1 knockout, FlxSGK1 mice were crossed with heterozygous dopamine transporter (DAT)-Cre mice (Jackson Laboratories, 006660). All mice were fully backcrossed to the c57Bl/6 background, and genotyping was performed at 3–4 weeks using the following primers for FlxSGK1 and DAT-Cre mouse lines.

SGK1 down: 5′-AAAGCTTATCTCAAACCCAAACCAA-3′.

SGK1 WT-Up: 5′-CTCATTCCAGACCGCTGACAAG-3′.

SGK1 mutant-Up: 5′-CTCAGTCTCTTTTGGGCTCTTT-3′.

DAT common: 5′-TGGCTGTTGGTGTAAAGTGG-3′.

DAT WT reverse: 5′-GGACAGGGACATGGTTGACT-3′.

DAT mutant reverse: 5′-CCAAAAGACGGCAATATGGT-3′.

Experiments utilized male and female mice (8–15 weeks). Mice were housed at 22–25 °C on a standard 12 h light–dark cycle with food and water ad libitum, and all behavior was measured during the light phase. All behavioral experiments were conducted on multiple cohorts of mice. Morphine preference, cocaine conditioned place preference, and sucrose preference were assessed in the same animals, with a 1-week recovery period between testing. The order of testing was counter-balanced for all experiments. Open field testing was similarly assessed in all animals but testing always occurred prior to any drug exposure. All experiments were approved by Michigan State University Institutional Animal Care and Use Committee (IACUC) and were carried out in accordance with the guidelines set in the Guide for the Care and Use of Laboratory Animals of the National Institutes of Health.

### Drugs

Cocaine hydrochloride was obtained from Sigma (#C5776). For all conditioned place preference experiments, cocaine was dissolved in 0.9% sterile saline. Male mice were conditioned with 12.5 mg/kg cocaine and females with 10 mg/kg cocaine. Because female rodents have been shown to form a conditioned preference at lower doses of cocaine compared to males^34,35^, a lower dose of drug was chosen for the females in order to achieve equivalent preference scores.

Morphine sulfate was generously provided by the NIDA Drug Supply Program. For all morphine two-bottle choice experiments, morphine was used at 0.05 mg/mL and dissolved in vehicle solution (0.2% sucrose in water). Subcutaneous sham and morphine pellets (25 mg) utilized in biochemical studies were also obtained from the NIDA Drug Supply Program.

### Viral-mediated gene transfer

Stereotaxic surgeries were completed following established procedures^6,40^. Briefly, mice were anesthetized (100 mg/kg ketamine, 10 mg/kg xylazine) and received bilateral intra-VTA infusions (0.5 μL) of rAAV2/CMV-Cre-recombinase (Cre)-GFP or rAAV2/TR-eGFP (University of North Carolina GTC Vector Core) at established coordinates (− 3.2 mm A/P, + 1.0 mm M/L, − 4.6 mm D/V, 7° angle). Mice were allowed to recover for at least 21 days before the start of behavioral experiments to allow for Cre-mediated gene deletion and the degradation of remaining SGK1 in infected cells. Following behavioral studies, viral targeting was confirmed by standard histological methods.

### Viral targeting

Mice were sacrificed and brains postfixed in 10% formalin for 3 days before cryo-preservation in 30% sucrose-phosphate buffered saline (PBS). Brains were then sliced into 30 μm sections and GFP labeling was used to confirm viral targeting. Mice with GFP expression outside of the VTA or with unilateral hits were excluded from analysis.

### Quantitative real-time PCR (qPCR)

At least 3 weeks following stereotaxic surgeries for VTA SGK1 KO or greater than 10 weeks of age for DA SGK1 KO, mice were sacrificed and VTA and NAc were microdissected and stored at -80 °C until processing. RNA was isolated and purified using RNAeasy micro-columns (Qiagen), and cDNA was created using a high capacity reverse transcription kit (Applied Biosystems). Changes in *sgk1* gene expression were determined via RT-PCR using Power SYBR green (CFX connect, BioRad) and published primers for SGK1 and GAPDH^57^:

SGK1 F: 5′-ATCGTGTTAGCTCCAAAGC-3′.

SGK1 R: 5′-GTCTGTGATCAGGCATAGC-3′.

GAPDH F: 5′-AGGTCGGTGTGAACGGATTTG-3′.

GAPDH R: 5′-TGTAGACCATGTAGTTGAGGTCA-3′.

Tyrosine hydroxylase (TH) and Cre-recombinase (Cre) were used to confirm tissue punch and viral targeting of the VTA:

TH F: 5′-CAGAGCAGGATACCAAGCAGG-3′.

TH R: 5′-CTCGAATACCACAGCCTCCAA-3′.

Cre F: 5′-GAACGAAAACGCTGGTTAGC-3′.

Cre R: 5′-CCCGGCAAAACAGGTAGTTA-3′.

All samples were run in triplicate and normalized to GAPDH before analysis using the ΔΔCt method.

### Western blots

Levels of phospho-SGK1 (pSer78) and phospho-NDRG (pNDRG, a readout of catalytic activity) were utilized as measures of altered SGK1 protein expression due to the lack of a reliable total SGK1 antibody. Because levels of SGK1 pSer78 and catalytic activity are low at baseline, mice were administered chronic morphine to robustly induce these biochemical changes^4^. Using published methods^58,59^, mice were subcutaneously implanted with either sham or morphine pellets (25 mg, generously provided by the NIDA Drug Supply Program) at least 3 weeks following stereotaxic surgeries for VTA SGK1 KO or greater than 10 weeks of age for DA SGK1 KO mice. Briefly, mice were anesthetized with isoflurane and implanted with a single subcutaneous sham or morphine pellet on days 1 and 3 before sacrifice on day 5. At the time of sacrifice, VTA and NAc were microdissected and stored at − 80 °C until processing.

For Western Blot analysis, tissue was sonicated in RIPA buffer containing protease and phosphatase inhibitors (Sigma) and centrifuged for 15 min at 20,000*g* (4 °C) before supernatants were collected and protein concentrations determined by Lowry Assay. Samples (20 μg) were loaded into precast SDS 4–15% gradient gels, electrophoresed, and transferred to PVDF membranes. Membranes were then blocked in 5% nonfat dairy milk (NFDM) in phosphate-buffered saline with 0.1% Tween-20 (PBS-T) for 1 h at 25 °C before an overnight incubation at 4 °C with primary antibodies in 5% bovine serum albumen (BSA) in PBS-T. The next day, membranes were washed with PBS-T, incubated in secondary antibody conjugated to horseradish peroxidase for 1 h at 25 °C in 5% NFDM in PBS-T, and washed again with PBS-T. Enhanced chemiluminescence was used to visualize protein bands. All data are normalized to the GAPDH loading control. Tyrosine hydroxylate (TH) and GFP expression were used to confirm tissue punch and viral targeting of the VTA. Primary antibodies were used as follows: phospho-Ser78 SGK1 (Cell Signaling, 5599 at 1:1,000), phospho-Thr346 NDRG (Cell Signaling, 3217 at 1:3,000), GAPDH (Cell Signaling, 2118 at 1:20,000), TH (Sigma, T1299 at 1:5,000), GFP (Invitrogen, A11122 at 1:1,000).

### Two-bottle voluntary choice (TBC) tasks

To assess voluntary drinking, mice were singly housed with access to two 50 mL conical tubes fitted with sipper tops following established procedures^40,57^. Briefly, every morning fluid consumption was measured, and bottle placement was switched to account for individual side bias. Mice that displayed a > 30% side/bottle preference during morphine preference were excluded from analysis. Mice were allowed to habituate to both bottles containing only water for 4 days, then water bottles were replaced for sucrose or morphine preference assessment for an additional 4 days. For sucrose preference, water bottles were replaced with bottles containing either 1% sucrose or water. For morphine preference, water bottles were replaced with bottles containing a 0.2% sucrose solution with either 0.05 mg/mL morphine sulfate or 0.01 mg/mL quinine (Sigma, 22640, bitter taste control)^60,61^. Drinking was measured to determine solution preference over time. Results are reported as percent preference (solution consumed/total fluid consumed × 100), average preference (4-day average of percent preference), and fluid intake (volume normalized to mouse body weight, 4-day average).

### Cocaine conditioned place preference (CPP)

Cocaine CPP was performed as previously described^6^. Briefly, mice were placed in the center of a 3-chambered CPP box (San Diego Instruments) and allowed to freely explore for 20 min to pretest for chamber bias. Mice with > 20% bias were excluded from analysis. During two conditioning days, mice were restricted to one chamber for 30 min and received a control (saline) i.p. injection in the morning and a cocaine injection (Sigma, 12.5 mg/kg for males or 10 mg/kg for females) in the opposite chamber in the afternoon. On test day, mice were placed in the center chamber and allowed to freely explore for 20 min. The CPP score was calculated as the time (sec) spent in the drug-paired minus saline-paired chamber. Locomotor activity was determined by total beam breaks during each session, and averages of conditioning sessions were used for analysis.

### Open field task

Mice were placed in a 38 cm × 38 cm arena and distance traveled was measured for 1 h using video-tracking software (TopScan Suite, CleverSys). Anxiety measures were calculated as the percent of time spent in the center and periphery of the area during the first 10 min of the session. Representative traces (Supplementary Fig. S3) display the first 10 min of locomotor activity.

### Body measurements

Body weights were taken once a week from 7 to 12 weeks of age, and body length was measured from the tip of the nose to the base of the tail at 12 weeks.

### Statistics

Full statistical analyses and results are listed in Supplementary Table S1. All statistical analyses were performed using GraphPad Prism, and all values are represented as mean ± SEM. An unpaired t test (two-tailed) was used to compare the means of two groups and a one-way analysis of variance (ANOVA) was used to compare the means of three groups, followed by a Tukey or Dunnett post-hoc test when appropriate. A two-way ANOVA was used when comparing two independent variables and a two-way ANOVA with repeating measures (RM) when two independent variables with repeated measures were compared, followed by a Tukey and Sidak or Dunnett post-hoc test when appropriate, respectively. Males and females were combined for biochemical validation, but always analyzed separately for behavior. Significance was defined as *p < 0.05 and **p < 0.01.

## Supplementary information

Supplementary Fig. 1

Supplementary Fig. 2

Supplementary Fig. 3

Supplementary Table

Supplementary legend

## Abbreviations

DA: Dopamine
VTA: Ventral tegmental area
SGK1: Serum- and glucocorticoid-inducible kinase 1
PKC: Protein kinase C
PDK1: 3-Phosphoinositide-dependent kinase 1
mTORC2: Mammalian target of rapamycin complex 2
ERK: Extracellular signal-regulated kinase
EGF: Epidermal growth factor
Na^+^: Sodium
ENaC: Epithelial Na^+^ channel
DAT: Dopamine transporter
FlxSGK1: Floxed SGK1
Cre: Cre-recombinase
NDRG: N-myc downstream regulated gene
NAc: Nucleus accumbens
TBC: Two-bottle choice
CPP: Conditioned place preference
KO: Knockout
