# Supplementary figures and images for: Behavioral effects of SGK1 knockout in VTA and dopamine neurons
Source: Sci Rep. 2020 Sep 8;10:14751. doi: 10.1038/s41598-020-71681-9 (PMC7478959; doi:10.1038/s41598-020-71681-9)

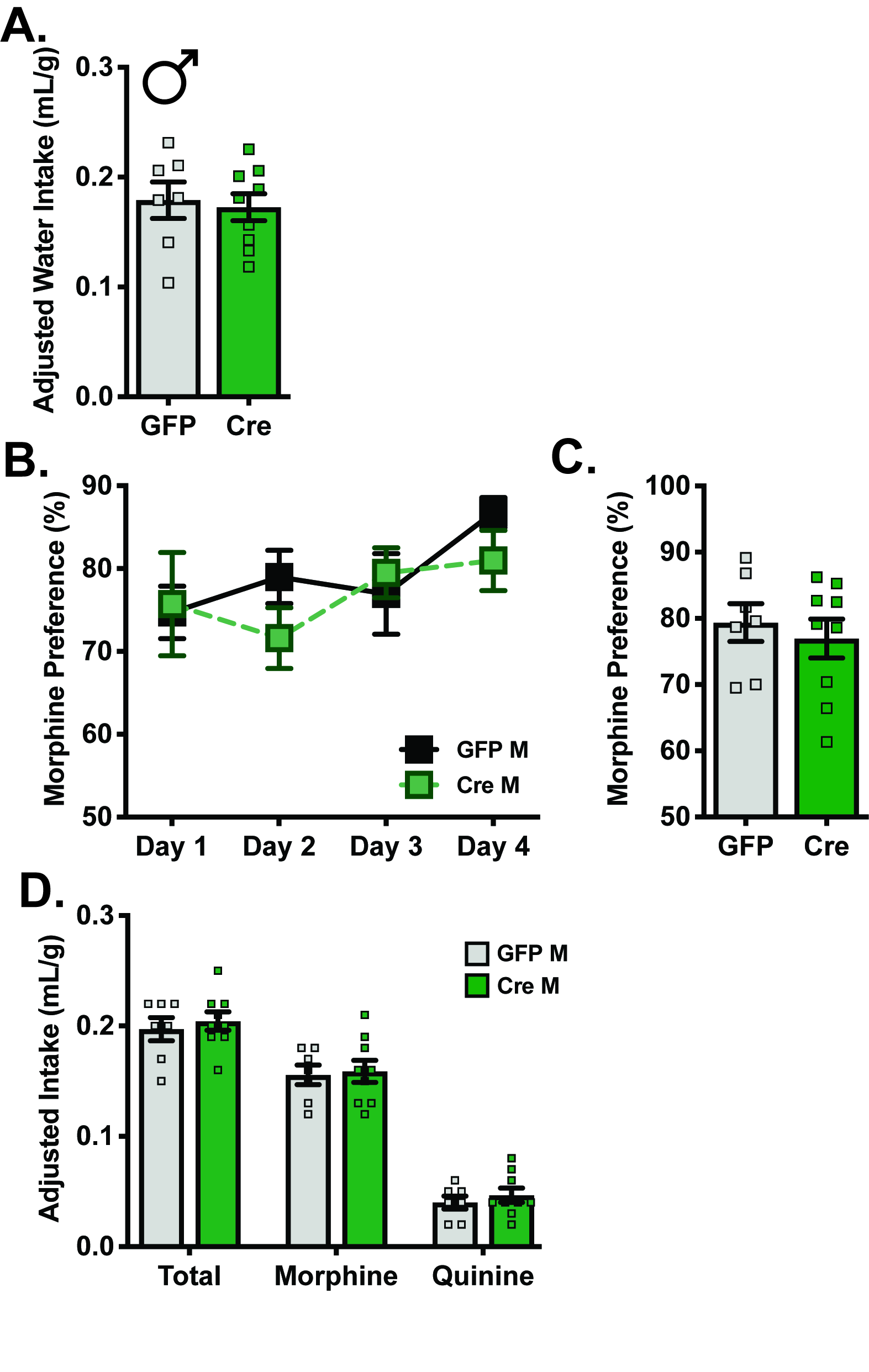

Supplement: Supplementary file 1 — Supplementary Fig. 1 [file 41598_2020_71681_MOESM1_ESM.tif]

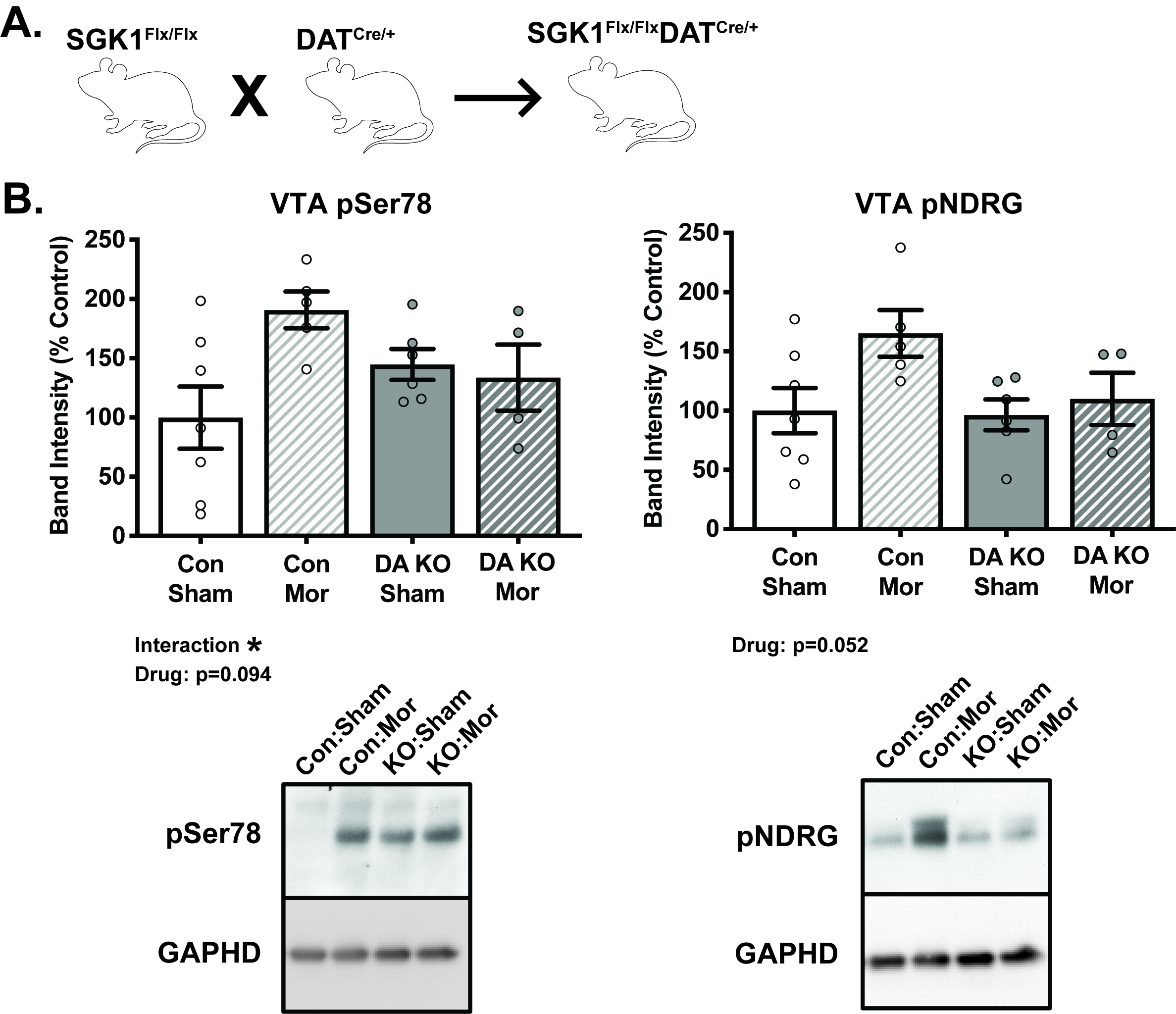

Supplement: Supplementary file 2 — Supplementary Fig. 2 [file 41598_2020_71681_MOESM2_ESM.tif]

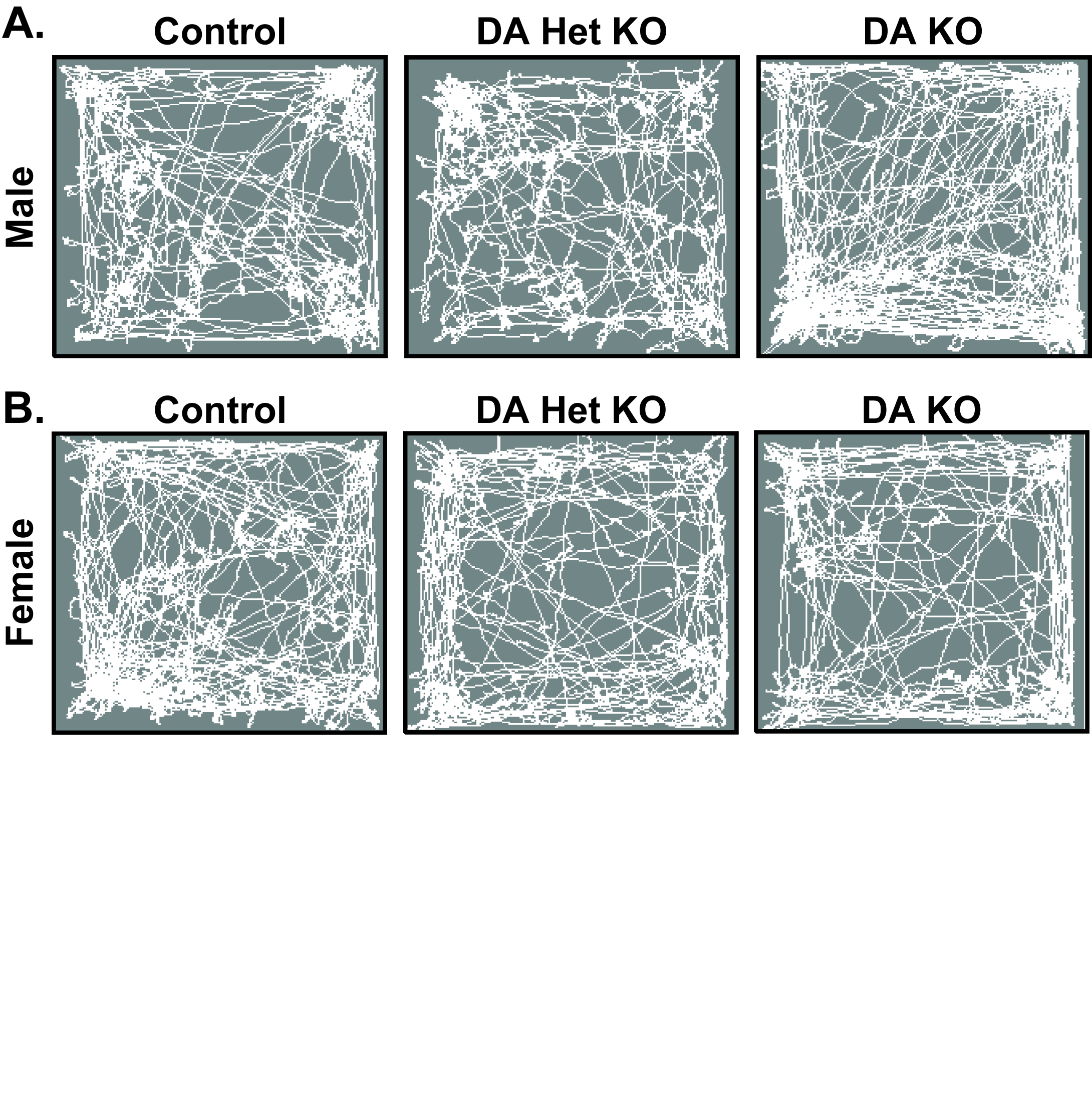

Supplement: Supplementary file 3 — Supplementary Fig. 3 [file 41598_2020_71681_MOESM3_ESM.tif]
