# Supplementary material for: Behavioral effects of SGK1 knockout in VTA and dopamine neurons
Source: Sci Rep. 2020 Sep 8;10:14751. doi: 10.1038/s41598-020-71681-9 (PMC7478959; doi:10.1038/s41598-020-71681-9)
Supplement: Supplementary file 4 — Supplementary Table [file 41598_2020_71681_MOESM4_ESM.pdf]

	Description	Sex	Group size	Statistical test	t-statistic	p-value	Main effect	f-statistic	p-value	Posthoc test	Posthoc comparison	p-value
**Figure 1. Establishment of VTA SGK1 knockdown mouse model.**												
**Fig. 1B**	VTA KD	Both	GFP: 14	Unpaired t-test	t=2.117, df=31	p= 0.0424						
	sgk1 mRNA		Cre: 19									
**Fig. 1C**	VTA KD											
	pSGK1 western	Both	GFP Sham: 4	Two-way ANOVA			Interaction	F (1, 11) = 0.9504	p=0.3506		GFP:Sham vs. Cre:Sham	p=0.9835
			GFP Mor: 4				Drug	F (1, 11) = 11.09	p=0.0067	Tukey	GFP:Sham vs. GFP:Morphine	p=0.0291
			Cre Sham: 2				Virus	F (1, 11) = 0.1678	p=0.6899		GFP:Sham vs. Cre:Morphine	p=0.1379
			Cre Mor: 5								Cre:Sham vs. GFP:Morphine	p=0.1421
											Cre:Sham vs. Cre:Morphine	p=0.4475
											GFP:Morphine vs. Cre:Morphine	p=0.6794
	VTA KD	Both	GFP Sham: 4	Two-way ANOVA			Interaction	F (1, 11) = 0.8146	p=0.3861			
	pNDRG western		GFP Mor: 4				Drug	F (1, 11) = 4.542	p=0.0565			
			Cre Sham: 2				Virus	F (1, 11) = 1.075	p=0.3221			
			Cre Mor: 5									
**Figure 2. Fluid intake and natural reward are not altered by VTA SGK1 knockdown.**												
**Fig. 2A**	VTA KD	Male	GFP: 7	Unpaired t-test	t=1.571, df=15	p=0.1370						
	Average water intake		Cre: 10									
		Female	GFP: 5	Unpaired t-test	t=0.3936, df=10	p=0.7021						
			Cre: 7									
**Fig. 2B**	VTA KD	Male	GFP: 7	Two-way ANOVA with RM			Time x Virus	F (3, 45) = 0.009888	p=0.9986			
	Daily sucrose preference		Cre: 10				Time	F (2.098, 31.48) = 13.64	p<0.0001			
							Virus	F (1, 15) = 1.321	p=0.2685			
		Female	GFP: 5	Two-way ANOVA with RM			Time x Virus	F (3, 30) = 4.277	p=0.0126	Sidak	GFP vs. Cre Day 1	p=0.6031
			Cre: 7				Time	F (2.294, 22.94) = 8.968	p=0.0009		GFP vs. Cre Day 2	p=0.5654
							Virus	F (1, 10) = 0.01378	p=0.9089		GFP vs. Cre Day 3	p=>0.9999
											GFP vs. Cre Day 4	p=0.4674
**In text**	VTA KD	Male	GFP: 7	Unpaired t-test	t=1.149, df=15	p=0.2685						
	Average sucrose preference		Cre: 10									
		Female	GFP: 5	Unpaired t-test	t=0.1161, df=10	p=0.9099						
			Cre: 7									
**Fig. 2C**	VTA KD	Male	GFP: 7	Unpaired t-test	t=0.9788, df=15	p=0.3432						
	Average total fluid intake		Cre: 10									
		Female	GFP: 5	Unpaired t-test	t=0.1052, df=10	p=0.9183						
			Cre: 7									
	Average sucrose intake	Male	GFP: 7	Unpaired t-test	t=1.109, df=15	p=0.2848						
			Cre: 10									
		Female	GFP: 5	Unpaired t-test	t=0.08385, df=10	p=0.9348						
			Cre: 7									
	Average water intake	Male	GFP: 7	Unpaired t-test	t=0.7222, df=15	p=0.4813						
			Cre: 10									
		Female	GFP: 5	Unpaired t-test	t=0.006383, df=10	p=0.9950						
			Cre: 7									

Figure 3. VTA SGK1 knockdown does not alter or morphine or cocaine reward behaviors.												
**Fig. 3A**	VTA KD	Male	GFP: 11	Two-way ANOVA with RM			Time x Virus	F (3, 84) = 2.257	p=0.0877			
	Daily morphine preference		Cre: 19				Time	F (3, 84) = 5.748	p=0.0013			
							Virus	F (1, 28) = 0.8411	p=0.3669			
		Female	GFP: 14	Two-way ANOVA with RM			Time x Virus	F (3, 93) = 1.247	p=0.2972			
			Cre: 19				Time	F (3, 93) = 3.887	p=0.0115			
							Virus	F (1, 31) = 4.027	p=0.0536			
**In text**	VTA KD	Male	GFP: 11	Unpaired t-test	t=0.9169, df=28	p=0.3670						
	Average morphine preference		Cre: 19									
		Female	GFP: 14	Unpaired t-test	t=2.006, df=31	p=0.0536						
			Cre: 19									
**Fig. 3B**	VTA KD	Male	GFP: 11	Unpaired t-test	t=1.281, df=28	p=0.2107						
	Average total fluid intake		Cre: 19									
		Female	GFP: 14	Unpaired t-test	t=0.05917, df=31	p=0.9532						
			Cre: 19									
	Average morphine intake	Male	GFP: 11	Unpaired t-test	t=1.502, df=28	p=0.1443						
			Cre: 19									
		Female	GFP: 14	Unpaired t-test	t=1.425, df=31	p=0.1641						
			Cre: 19									
	Average quinine intake	Male	GFP: 11	Unpaired t-test	t=0.4314, df=28	p=0.6694						
			Cre: 19									
		Female	GFP: 14	Unpaired t-test	t=1.913, df=31	p=0.0650						
			Cre: 19									
**Fig. 3C**	VTA KD	Male	GFP: 18	Unpaired t-test	t=0.8243, df=35	p=0.4154						
	CPP post-test scores		Cre: 19									
		Female	GFP: 13	Unpaired t-test	t=0.1844, df=27	p=0.8551						
			Cre: 16									
**Fig. 3D**	VTA KD	Male	GFP: 18	Two-way ANOVA			Drug x Virus	F (1, 70) = 0.01100	p=0.9168		Saline:GFP vs. Saline:Cre	p=0.9981
	CPP conditioning locomotor activity		Cre: 19				Drug	F (1, 70) = 21.17	p<0.0001	Tukey	Saline:GFP vs. Cocaine:GFP	p=0.013
							Virus	F (1, 70) = 0.1245	p=0.7253		Saline:GFP vs. Cocaine:Cre	p=0.0044
											Saline:Cre vs. Cocaine:GFP	p=0.0189
											Saline:Cre vs. Cocaine:Cre	p=0.0065
											Cocaine:GFP vs. Cocaine:Cre	p=0.9882
		Female	GFP: 13	Two-way ANOVA			Drug x Virus	F (1, 54) = 0.1042	p=0.7481		Saline :GFP vs. Saline :Cre	p=0.9999
			Cre: 16				Drug	F (1, 54) = 15.47	p=0.0002	Tukey	Saline :GFP vs. Cocaine:GFP	p=0.0834
							Virus	F (1, 54) = 0.1825	p=0.6709		Saline :GFP vs. Cocaine:Cre	p=0.0165
											Saline :Cre vs. Cocaine:GFP	p=0.0747
											Saline :Cre vs. Cocaine:Cre	p=0.0127
											Cocaine:GFP vs. Cocaine:Cre	p=0.9513

Figure 4. Body weight and locomotor activity, but not anxiety, are altered by DA SGK1 KO in both male and female mice.

**Fig. 4A**	DA SGK1 KO	Males	Con: 23	Two-way ANOVA with RM			Time x Genotype	F (10, 195) = 3.091	p=0.0011	Sidak	7 Control M vs. Het KO M	p=0.1863
	Weight		Het: 7				Time	F (5, 195) = 185.4	p<0.0001		7 Control M vs. KO M	p=0.2526
			KO: 12				Genotype	F (2, 39) = 3.094	p=0.0566		8 Control M vs. Het KO M	p=0.0628
							Subject	F (39, 195) = 60.78	p<0.0001		8 Control M vs. KO M	p=0.0857
											9 Control M vs. Het KO M	p=0.2404
											9 Control M vs. KO M	p=0.0243
											10 Control M vs. Het KO M	p=0.0602
											10 Control M vs. KO M	p=0.0451
											11 Control M vs. Het KO M	p=0.1339
											11 Control M vs. KO M	p=0.0858
											12 Control M vs. Het KO M	p=0.7887
											12 Control M vs. KO M	p=0.0299
		Females	Con: 19	Two-way ANOVA with RM			Time x Genotype	F (10, 190) = 5.275	p<0.0001	Sidak	7 Control F vs. Het KO F	p=0.9999
			Het: 9				Time	F (5, 190) = 48.48	p<0.0001		7 Control F vs. KO F	p=0.218
			KO: 13				Genotype	F (2, 38) = 3.743	p=0.0328		8 Control F vs. Het KO F	p=0.395
							Subject	F (38, 190) = 27.68	p<0.0001		8 Control F vs. KO F	p=0.0639
											9 Control F vs. Het KO F	p=0.169
											9 Control F vs. KO F	p=0.0263
											10 Control F vs. Het KO F	p=0.0478
											10 Control F vs. KO F	p=0.1138
											11 Control F vs. Het KO F	p=0.0036
											11 Control F vs. KO F	p=0.0643
											12 Control F vs. Het KO F	p=0.0005
											12 Control F vs. KO F	p=0.0217
**In text**	DA SGK1 KO	Male	Con: 7	Unpaired t-test	t=1.629, df=15	p=0.1241						
	Body length at 12 weeks of age		KO: 10									
**Fig. 4B**	DA SGK1 KO	Male	Con: 18	One-way ANOVA				F (2, 34) = 9.339	p=0.0006	Dunnett	Con M vs. Het KO M	p=0.1354
	Locomotor activity		Het: 7								Con M vs. KO M	p=0.0003
			KO: 12									
		Female	Con: 19	One-way ANOVA				F (2, 38) = 3.074	p=0.0579			
			Het: 9									
			KO: 13									

**Fig. 4C**	DA SGK1 KO	Male	Con: 18	Two-way ANOVA with RM	Time x Genotype	F (22, 374) = 2.831	p<0.0001	Dunnett	5 Con M vs. Het KO M	p=0.7396
	Habituation curve		Het: 7		Time	F (11, 374) = 37.28	p<0.0001		5 Con M vs. KO M	p<0.0001
			KO: 12		Genotype	F (2, 34) = 9.373	p=0.0006		10 Con M vs. Het KO M	p=0.1735
					Subject	F (34, 374) = 15.17	p<0.0001		10 Con M vs. KO M	p<0.0001
									15 Con M vs. Het KO M	p=0.0851
									15 Con M vs. KO M	p<0.0001
									20 Con M vs. Het KO M	p=0.1808
									20 Con M vs. KO M	p=0.0006
									25 Con M vs. Het KO M	p=0.264
									25 Con M vs. KO M	p=0.0006
									30 Con M vs. Het KO M	p=0.1567
									30 Con M vs. KO M	p=0.0083
									35 Con M vs. Het KO M	p=0.0535
									35 Con M vs. KO M	p=0.0011
									40 Con M vs. Het KO M	p=0.0164
									40 Con M vs. KO M	p=0.0021
									45 Con M vs. Het KO M	p=0.0754
									45 Con M vs. KO M	p=0.0138
									50 Con M vs. Het KO M	p=0.697
									50 Con M vs. KO M	p=0.2143
									55 Con M vs. Het KO M	p=0.9183
									55 Con M vs. KO M	p=0.2632
									60 Con M vs. Het KO M	p=0.4356
									60 Con M vs. KO M	p=0.0941
		Female	Con: 19	Two-way ANOVA with RM	Time x Genotype	F (33, 462) = 0.9163	p=0.6041			
			Het: 9		Time	F (5.135, 215.7) = 31.08	p<0.0001			
			KO: 13		Genotype	F (3, 42) = 1.978	p=0.1319			
					Subject	F (42, 462) = 19.23	p<0.0001			
**Fig. 4D**	DA SGK1 KO	Male	Center	One-way ANOVA		F (2, 34) = 0.8585	p=0.4328			
	Anxiety in open field		Con: 18							
			Het: 7							
			KO: 12							
		Male	Peripheral	One-way ANOVA		F (2, 34) = 0.8592	p=0.4325			
			Con: 18							
			Het: 7							
			KO: 12							
		Female	Center	One-way ANOVA		F (2, 39) = 0.4309	p=0.6530			
			Con: 19							
			Het: 9							
			KO: 13							
		Female	Peripheral	One-way ANOVA		F (2, 39) = 0.4303	p=0.6533			
			Con: 19							
			Het: 9							
			KO: 13							

**Figure 5. Fluid intake and preference for natural reward is not altered by VTA DA KO.**									
**Fig. 5A**	DA SGK1 KO<br>Average water intake	Male	Con: 10<br>KO: 5	Unpaired t-test	t=0.1311, df=13	p=0.8977			
		Female	Con: 7<br>KO: 8	Unpaired t-test	t=0.3296, df=13	p=0.7470			
**Fig. 5B**	DA SGK1 KO<br>Daily sucrose preference	Male	Con: 10<br>KO: 5	Two-way ANOVA with RM			Time x Genotype	F (3, 39) = 0.01410	p=0.9977
							Time	F (3, 39) = 2.459	p=0.0772
							Genotype	F (1, 13) = 1.157	p=0.3017
							Subject	F (13, 39) = 1.217	p=0.3046
		Female	Con: 7<br>KO: 8	Two-way ANOVA with RM			Time x Genotype	F (3, 39) = 1.045	p=0.3837
							Time	F (3, 39) = 2.450	p=0.0780
							Genotype	F (1, 13) = 3.156	p=0.0990
							Subject	F (13, 39) = 0.6906	p=0.7601
**In text**	VTA KO<br>Average sucrose preference	Male	Con: 10<br>KO: 5	Unpaired t-test	t=1.075, df=13	p=0.3018			
		Female	Con: 7<br>KO: 8	Unpaired t-test	t=1.776, df=13	p=0.0991			
**Fig. 5C**	DA SGK1 KO<br>Average total fluid intake	Male	Con: 10<br>KO: 5	Unpaired t-test	t=0.5621, df=13	p=0.5836			
		Female	Con: 7<br>KO: 8	Unpaired t-test	t=0.3539, df=13	p=0.7291			
	Average sucrose intake	Male	Con: 10<br>KO: 5	Unpaired t-test	t=1.383, df=13	p=0.1901			
		Female	Con: 7<br>KO: 8	Unpaired t-test	t=1.183, df=13	p=0.2580			
	Average water intake	Male	Con: 10<br>KO: 5	Unpaired t-test	t=0.7019, df=13	p=0.4951			
		Female	Con: 7<br>KO: 8	Unpaired t-test	t=1.507, df=13	p=0.1557			
**Figure 6. DA SGK1 KO does not alter morphine or cocaine reward.**									
**Fig. 6A**	DA SGK1 KO<br>Daily morphine preference	Male	Con: 12<br>KO: 8	Two-way ANOVA with RM			Time x Genotype	F (3, 54) = 0.5270	p=0.6656
							Time	F (3, 54) = 1.592	p=0.2020
							Genotype	F (1, 18) = 0.5089	p=0.4847
							Subject	F (18, 54) = 1.848	p=0.0427
		Female	Con: 12<br>KO: 9	Two-way ANOVA with RM			Time x Genotype	F (3, 57) = 0.2738	p=0.8441
							Time	F (3, 57) = 1.038	p=0.3829
							Genotype	F (1, 19) = 0.008160	p=0.9290
							Subject	F (19, 57) = 2.210	p=0.0111
**In text**	VTA KO<br>Average morphine preference	Male	Con: 12<br>KO: 8	Unpaired t-test	t=0.7137, df=18	p=0.4846			
		Female	Con: 12<br>KO: 9	Unpaired t-test	t=0.09002, df=19	p=0.9292			

**Fig. 6B**	DA SGK1 KO<br>Average total fluid intake	Male	Con: 12<br>KO: 8	Unpaired t-test	t=0.8533, df=18	p=0.4047						
		Female	Con: 12<br>KO: 9	Unpaired t-test	t=0.4895, df=19	p=0.6301						
	Average morphine intake	Male	Con: 12<br>KO: 8	Unpaired t-test	t=0.5629, df=18	p=0.5804						
		Female	Con: 12<br>KO: 9	Unpaired t-test	t=0.4213, df=19	p=0.6783						
	Average quinine intake	Male	Con: 12<br>KO: 8	Unpaired t-test	t=0.8259, df=18	p=0.4197						
		Female	Con: 12<br>KO: 9	Unpaired t-test	t=0.1430, df=19	p=0.8878						
**Fig. 6C**	DA SGK1 KO<br>CPP post-test scores	Male	Con: 20<br>KO: 11	Unpaired t-test	t=0.9219, df=29	p=0.3642						
		Female	Con: 17<br>KO: 11	Unpaired t-test	t=0.3210, df=26	p=0.7508						
**Fig. 6D**	DA SGK1 KO<br>CPP conditioning locomotor activity	Male	Con: 20<br>KO: 11	Two-way ANOVA			Drug x Genotype	F (1, 58) = 0.1445	p=0.7052		Saline:Con M vs. Saline:KO M	p=0.8072
							Drug	F (1, 58) = 17.42	p=0.0001	Tukey	Saline:Con M vs. Cocaine:Con M	p=0.0018
							Genotype	F (1, 58) = 0.7855	p=0.3791		Saline:Con M vs. Cocaine:KO M	p=0.0038
											Saline:KO M vs. Cocaine:Con M	p=0.1041
											Saline:KO M vs. Cocaine:KO M	p=0.0961
											Cocaine:Con M vs. Cocaine:KO M	p=0.9841
		Female	Con: 17<br>KO: 11	Two-way ANOVA			Drug x Genotype	F (1, 52) = 0.05082	p=0.8225		Saline:Con F vs. Saline:KO F	p=0.9983
							Drug	F (1, 52) = 18.96	p<0.0001	Tukey	Saline:Con F vs. Cocaine:Con F	p=0.0094
							Genotype	F (1, 52) = 0.0001199	p=0.9913		Saline:Con F vs. Cocaine:KO F	p=0.0173
											Saline:KO F vs. Cocaine:Con F	p=0.0166
											Saline:KO F vs. Cocaine:KO F	p=0.0245
											Cocaine:Con F vs. Cocaine:KO F	p=0.9987
**Supplemental Figure S1. Heterozygous VTA knockdown does not alter morphine preference in males.**												
**Fig. S1A**	Heterozygous VTA KO<br>Average water intake	Male	GFP: 7<br>Cre: 9	Unpaired t-test	t=0.3181, df=14	p=0.7551						
**Fig. S1B**	Heterozygous VTA KO<br>Daily morphine preference	Male	GFP: 7<br>Cre: 9	Two-way ANOVA with RM			Time x Virus	F (3, 42) = 1.142	p=0.3434			
							Time	F (3, 42) = 3.131	p=0.0355			
							Virus	F (1, 14) = 0.3306	p=0.5745			
							Subject	F (14, 42) = 3.318	p=0.0013			
**Fig. S1C**	Heterozygous VTA KO<br>Average morphine preference	Male	GFP: 7<br>Cre: 9	Unpaired t-test	t=0.5744, df=14	p=0.5748						
**Fig. S1D**	Heterozygous VTA KO<br>Average total fluid intake	Male	GFP: 7<br>Cre: 9	Unpaired t-test	t=0.5543, df=14	p=0.5881						
	Average morphine intake	Male	GFP: 7<br>Cre: 9	Unpaired t-test	t=0.2283, df=14	p=0.8227						
	Average quinine intake	Male	GFP: 7<br>Cre: 9	Unpaired t-test	t=0.7462, df=14	p=0.4679						

Supplemental Figure S2. VTA SGK1 catalytic activity and phosphorylation in DA SGK1 KO mice.										
**Fig. S2A**	DA SGK KO	Both	Con Sham: 7	Two-way ANOVA	Interaction	F (1, 18) = 5.109	p=0.0364	Tukey	Con:Sham vs. KO:Sham	p=0.4275
	pSer78 western		Con Mor: 5		Drug	F (1, 18) = 3.120	p=0.0943		Con:Sham vs. Con:Morphine	p=0.0355
			KO Sham: 6		Genotype	F (1, 18) = 0.07555	p=0.7866		Con:Sham vs. KO:Morphine	p=0.7305
			KO Mor: 4						KO:Sham vs. Con:Morphine	p=0.4755
									KO:Sham vs. KO:Morphine	p=0.9868
									Con:Morphine vs. KO:Morphine	p=0.3791
**Fig. S2B**	DA SGK KO	Both	Con Sham: 7	Two-way ANOVA	Interaction	F (1, 18) = 1.864	p=0.1890			
	pNDRG western		Con Mor: 5		Drug	F (1, 18) = 4.321	p=0.0522			
			KO Sham: 6		Genotype	F (1, 18) = 2.415	p=0.1376			
			KO Mor: 4							
