# Supplementary material for: Behavioral effects of SGK1 knockout in VTA and dopamine neurons
Source: Sci Rep. 2020 Sep 8;10:14751. doi: 10.1038/s41598-020-71681-9 (PMC7478959; doi:10.1038/s41598-020-71681-9)
Supplement: Supplementary file 5 — Supplementary legend [file 41598_2020_71681_MOESM5_ESM.docx]

**Supplemental Figure S1. Heterozygous VTA knockdown does not alter morphine preference in males.**

**S1A.** No change in water intake was observed in males with heterozygous VTA SGK1 knockdown compared to GFP controls (n=7-9, unpaired t-test).

**S1B.** Daily morphine preference in a TBC task was not changed by heterozygous knockdown of SGK1 in the VTA of males (n=7-9, two-way ANOVA with repeated measures).

**S1C.** Average morphine preference was similarly unaffected in knockdown mice compared to GFP controls (n=7-9, unpaired t-test).

**S1D.** Heterozygous SGK1 knockdown did not alter fluid intake during the morphine TBC task (n=7-9, unpaired t-test).

Supplemental Figure S2. VTA SGK1 catalytic activity and phosphorylation in DA SGK1 KO mice.

A. Representation of breeding strategy for generating the DA SGK1 KO mice.

B. Following morphine or sham treatment in DA SGK1 KO and control mice, there was a significant interaction of drug and DA SGK1 KO on VTA phospho-SGK1 (pSer78) while no significant effects were observed on VTA pNDRG (pSer78 n=4-7, two-way ANVOA followed by a Tukey post-hoc test, drug x genotype interaction: p=0.036; pNDRG n=4-7, two-way ANVOA).

Supplemental Figure S3. Representative traces for open field locomotor activity of DA SGK1 KO mice.

A. Representative locomotor traces during the first 10 minutes of open field testing in male control, heterozygous DA SGK1 KO, and homozygous DA SGK1 KO mice.

B. Representative locomotor traces for female control, heterozygous DA SGK1 KO, and homozygous DA SGK1 KO mice during the first 10 minutes of open field testing.

Supp. Data Table 1: Table of complete statistical analyses
